# A Simple and Rapid Procedure for the Detection of Genes Encoding Shiga Toxins and Other Specific DNA Sequences

**DOI:** 10.3390/toxins7114745

**Published:** 2015-11-13

**Authors:** Bożena Nejman-Faleńczyk, Sylwia Bloch, Aleksandra Januszkiewicz, Alicja Węgrzyn, Grzegorz Węgrzyn

**Affiliations:** 1 Depratment of Molecular Biology, University of Gdańsk, Wita Stwosza 59, 80-308 Gdańsk, Poland; E-Mails: sylwia.bloch@biol.ug.edu.pl (S.B.); grzegorz.wegrzyn@biol.ug.edu.pl (G.W.); 2 Department of Bacteriology, National Institute of Public Health-Public Institute of Hygiene, 24 Chocimska Street, 00-791 Warsaw, Poland; E-Mail: ajanuszkiewicz@pzh.gov.pl; 3 Laboratory of Molecular Biology (affiliated with the University of Gdansk), Institute of Biochemistry and Biophysics, Polish Academy of Sciences, Wita Stwosza 59, 80-308 Gdansk, Poland; E-Mail: alicja.wegrzyn@biol.ug.edu.pl

**Keywords:** DNA amplification, detection methods, PCR product detection, shiga toxin-producing *Escherichia coli*, *stx* genes

## Abstract

A novel procedure for the detection of specific DNA sequences has been developed. This procedure is based on the already known method employing PCR with appropriate primers and a sequence-specific DNA probe labeled with the fluorescent agent 6-carboxylfluorescein (FAM) at the 5′ end and the fluorescence quencher BHQ-1 (black hole quencher) at the 3′ end. However, instead of the detection of the fluorescence signal with the use of real-time PCR cyclers, fluorescence/luminescence spectrometers or fluorescence polarization readers, as in all previously-reported procedures, we propose visual observation of the fluorescence under UV light directly in the reaction tube. An example for the specific detection of the Shiga toxin-producing *Escherichia coli* (STEC) strains, by detecting Shiga toxin genes, is demonstrated. This method appears to be specific, simple, rapid and cost effective. It may be suitable for use in research laboratories, as well as in diagnostic units of medical institutions, even those equipped only with a thermocycler and a UV transilluminator, particularly if rapid identification of a pathogen is required.

## 1. Introduction

Polymerase chain reaction (PCR) is widely used in both basic research and biotechnological or medical applications, to such an extent that it is considered a very common technique for most genetic analyses. Although the reaction conditions can be optimized to get the best specificity and efficiency, the detection of specific PCR products is perhaps the most rate-limiting step in the vast majority of PCR-based techniques. A number of methods for the detection of such products has been proposed and reported; among them, gel electrophoresis and various hybridization procedures (e.g., dot-blot, Southern blot, reverse hybridization, DNA enzyme immunoassay, *etc.*) predominate [1,2,3,4,5,6,7,8,9]. A wide range of commercialized diagnostic methods is based on a combination of PCR and gel electrophoresis. The main disadvantage of such a combination is the lack of the possibility to distinguish between specific and non-specific amplification products; thus, many laboratories use additional techniques to confirm the specificity of the amplified PCR product [10,11]. Despite the fact that different methods could improve the sensitivity or specificity of the detection, such procedures, which are performed after the DNA amplification reaction, are time consuming, and usually require specialized and expensive equipment [12,13,14,15]. This could be a significant problem, particularly if rapid detection of a specific signal is crucial and the availability of sophisticated equipment is problematic, like in the case of diagnostic procedures performed at local (small) medical laboratories. Therefore, we aimed to develop a simple and rapid procedure for the detection of specific PCR products, which could be used even at a basically-equipped laboratory.

**Figure 1:**
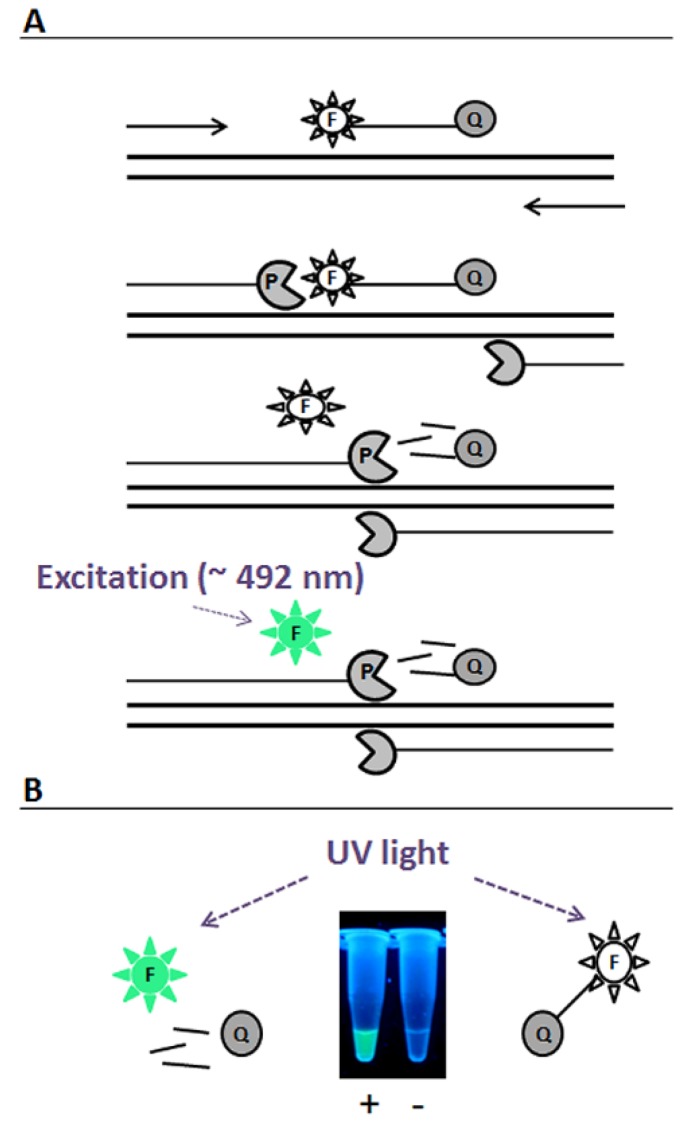
(**A**) A scheme for the procedure of the detection of specific DNA sequences reported in this work. The procedure is based on the already known method employing PCR with DNA polymerase (designated as P), appropriate primers and a sequence-specific DNA probe labeled with the fluorescent agent FAM (designated as F) at the 5′ end and the fluorescence quencher BHQ-1 (black hole quencher; designated as Q) at the 3′ end. (**B**) Visual observation of the fluorescence under UV light at 302 nm directly in the reaction tube is proposed, instead of detection of the fluorescence signal with the use of real-time PCR cyclers, fluorescence/luminescence spectrometers or fluorescence polarization readers and as an alternative to the gel electrophoresis technique. PCR reactions with (+) and without (−) targeted pathogenic DNA were analyzed.

One of the previousy described methods for the detection of the presence of certain DNA sequences is based on the PCR reaction with appropriate primers and specific probes labeled with the fluorescent agent 6-carboxylfluorescein (FAM) at the 5′ end and the fluorescence quencher BHQ-1 (black hole quencher) at the 3′ end. FAM is a fluorescein derivative with an excitation peak at 492 nm and an emission peak at 517 nm [16,17,18,19]. According to this method, fluorescence may appear only if the probe hybridizes to the target DNA region and is subsequently degraded by the Taq DNA polymerase extending a specific primer designed to amplify a DNA fragment that includes the probe binding site. In such a case, FAM is no longer located in the proximity of BHQ-1; thus, its fluorescence is not quenched, and light might be emitted after excitation at the appropriate wavelength [16,18,19] (Figure 1A). However, in previously-reported procedures employing this phenomenon, the fluorescence emitted by FAM was detected using light excitation sources of a wavelength in the range of the visible spectrum, near the excitation maximum of FAM (~492 nm), such as lasers and diodes in real-time PCR cyclers, fluorescence/luminescence spectrometers or fluorescence polarization readers [18,20,21], which made them relatively expensive. Thus, we asked if it is possible to simplify the detection procedure, to make it inexpensive and rapid, while still keeping its sensitivity at an acceptable level. We would like to propose a different detection manner of fluorescence emitted by FAM, released from the BHQ-1 influence, based on the excitation with light outside the visible range, *i.e.*, ultraviolet light, directly in the reaction tube (Figure 1B). The modification we aimed to introduce allows confirmation of the presence of the target DNA, amplified during PCR reaction in the test tube, without the need of running agarose gel electrophoresis. Such a method would be particularly desirable in detection of pathogenic microorganisms, especially those requiring rapid diagnostic methods.

Among the pathogenic microorganisms requiring rapid detection are Shiga toxin-producing *Escherichia coli* (STEC), also called verotoxic *E. coli*, and thus, we decided to use these bacteria in testing the new procedure. STEC is comprised of a group of bacterial strains belonging to the usually non-pathogenic species (*E. coli*), which have gained genes turning them into serious pathogens [22]. The best known examples are genes encoding Shiga toxins (*stx*), located on lambdoid prophages called Shiga toxin-converting (abbreviated Stx) prophages, which are responsible for their transmission between *E. coli* species [23]. People infected with STEC bacteria may have some serious symptoms, which are especially severe in the case of a subset of STEC, called enterohemorrhagic *E. coli* (EHEC). These symptoms include bloody diarrhea, which can progress to hemorrhagic colitis or hemolytic uremic syndrome [24]. Importantly, treatment of patients infected with STEC is often problematic, as many antibiotics stimulate the induction of the Shiga toxin-converting prophages, the event that is necessary for the efficient expression of Shiga toxin genes, thus enhancing the severity of the disease symptoms [25]. The importance of STEC-dependent health problems can be highlighted by the results of the recent outbreak that occurred in Germany in 2011, when out of over 4000 cases of symptomatic infections, over 50 patients died [26,27,28,29].

Since treatment of patients infected with STEC should be considerably different from that recommended in the case of other infectious diseases, it was stressed that proper and rapid identification of the pathogen is crucial when production of Shiga toxin is suspected [30]. This implies that rapid diagnostic procedures are required. However, contrary to early predictions, serological tests are not adequate for STEC detection, as Shiga toxins can be produced by various *E. coli* serotypes [23]. Therefore, DNA-based methods appear to be mandatory for unequivocal identification of STEC. Nevertheless, although different sophisticated methods for highly precise and even quantitative determination of the presence of these bacteria have become available, as summarized recently [30], the vast majority of them are either time consuming, or expensive, or both. Therefore, such methods may be excellent for more detailed analyses, but perhaps unavailable or even useless for rapid detection of STEC infections in small laboratories, like those located in many provincial hospitals. Hence, we assumed that the development of a simple, specific and quick method for rapid detection of STEC may be an example of the potential usefulness of the novel procedure described in this report. 

## 2. Results and Discussion

**Figure 2:**
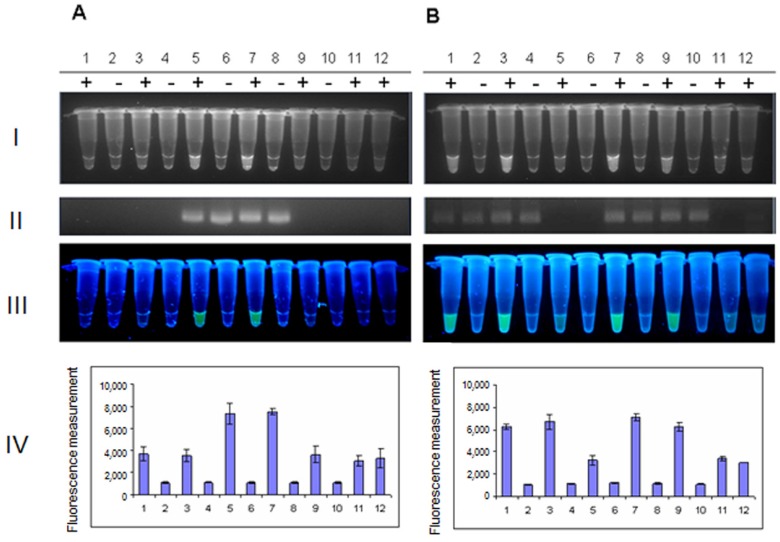
The analysis of Shiga toxin-producing Escherichia coli (STEC) strains: 286/00 (*stx1*^−^ *stx2*^+^) (Lanes 1–2), 201/01 (*stx1*^−^ *stx2*^+^) (Lanes 3–4), 319/01 (*stx1*^+^ *stx2*^−^) (Lanes 5–6), 44/02 (*stx1*^+^ *stx2*^+^) (Lanes 7–8) and 174/03 (*stx1*^−^ *stx2*^+^) (Lanes 9–10) for the presence of the gene encoding (**A**) Shiga toxin 1 and (**B**) Shiga toxin 2, using PCR with a FAM- and BHQ-1-labelled probe (+) or without such a probe (−). Detection of the signal from the tox1probe or tox2probe, complementary to the gene encoding Shiga toxin 1 or Shiga toxin 2, respectively, was performed using: (I) the gel documentation system Gel Doc XR, Bio-Rad; (II) analysis of PCR products by agarose gel electrophoresis; (III) a UV transilluminator; or (IV) measurement of the fluorescence signal at 485/535 nm (means of three experiments ± SD) in a plate reader. The control reaction performed without the DNA template (Lane 11) and the reaction performed with genomic DNA of the *E. coli* MG1655 strain, which does not contain genes coding for Shiga toxins 1 and 2 (Lane 12), are also shown.

The specific PCR amplification of fragments of genes encoding Shiga toxins 1 and 2, in the presence of probes labeled with the fluorescent agent FAM and the fluorescence quencher BHQ-1, was performed according to a previously published method [16,17]. In the procedure described in this report, we propose to detect the specific fluorescent signal by a simple observation of the reaction tube over a UV transilluminator (Figure 1B). We found that detection of such a signal is unequivocal, and the signal is specific. When 20 different *E. coli* strains (Table 1) were investigated, the fluorescence occurred only when genomes of the tested bacteria contained the target gene(s) (either *stx1*, or *stx2*, or both). We observed 100% compatibility between results obtained by the proposed method and results presented in Table 1, obtained by methods determined as “gold standards”. Figure 2 shows examples of these experiments, with controls, including analyses of PCR products by agarose gel electrophoresis and the results of measurement of the fluorescence signal generated at 485/535 nm, during each PCR reaction. The fluorescence light, emitted in response to UV exposure, was compatible with the agarose gel band patterns and measurements of the fluorescence signal in a plate reader. As described previously [31], the FAM-BHQ-labelled probe, which is not degraded during the PCR reaction (because of the lack of the target DNA), exhibits some level of background fluorescence. In negative controls, the background fluorescence comes from the unhybridized probe itself and is typical for such linear probes because of the relatively long distance between the fluorescence reporter and quencher, which results in inefficient quenching. The level of the background fluorescence can be detected by sensitive fluorescence detectors, like real-time PCR cyclers or plate readers, as in this particular case (Figure 2A, Row IV, Columns 1, 3, 9, 11, 12 and Figure 2B, Row IV, Columns 5, 11, 12). Interestingly, as indicated in Figure 2 (A and B, Row I), in the proposed method, the level of the background fluorescence is low enough and not visible during the observation of the tubes under UV light.

**Table 1:** *Escherichia coli* strains.

No.	Strain	Serotype	*Stx1* Status	*Stx2* Status
1	286/00	O157	−	+
2	44/02	O157	+	+
3	174/03	O157	−	+
4	49/04	O157	+	+
5	365/05	O157	+	+
6	206/06	O157	+	+
7	443/07	O157	+	+
8	474/07	O157	+	+
9	9/08	O157	−	+
10	221/08	O157	+	+
11	371/08	O157	−	+
12	171/09	O157	−	+
13	74/10	O157	−	+
14	245/10	O111	−	+
15	251/10	O157	+	+
16	201/01	O26	−	+
17	319/01	O26	+	−
18	571	O157	+	+
19	EDL933W	O157	+	+
20	MG1655	K12	−	−

**Figure 3:**
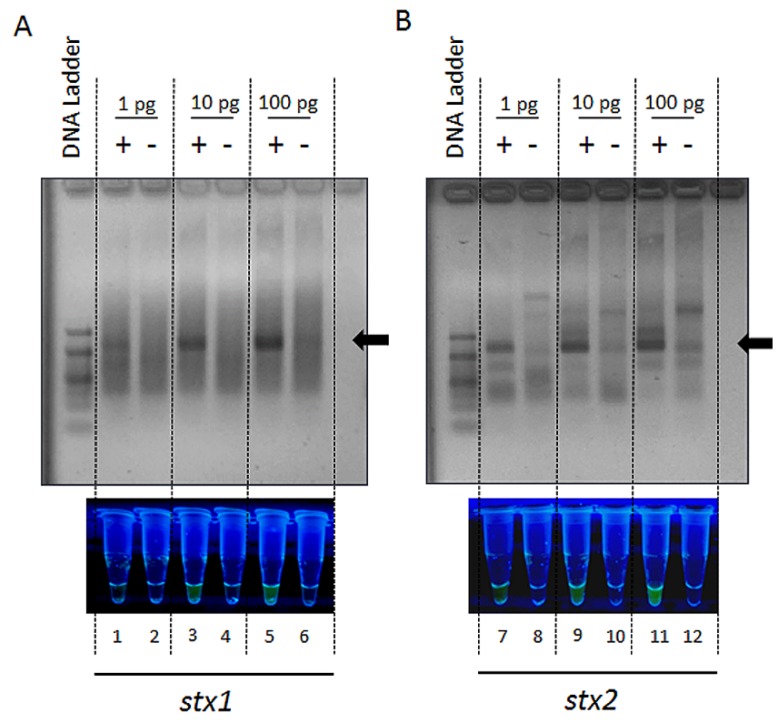
The analysis of STEC *stx*-positive strain (+) EDL933W and the *stx*-negative (−) strain MG1655 of *E. coli* K12 bacteria for the presence of the genes encoding Shiga toxins 1 and 2 and the occurrence of nonspecific PCR products using PCR with a FAM- and BHQ-1-labelled probe. Detection of the signal from the tox1probe (**A**) or tox2probe (**B**), complementary to the genes encoding Shiga toxin 1 or Shiga toxin 2, respectively, was performed by analysis of PCR products by agarose gel electrophoresis and using a UV transilluminator. Black arrows indicate the size of the target PCR products: 196 bp for *stx1* and 211 bp for *stx2*.

Due to the fact that phages bearing *stx1* and *stx2* genes can be transmitted to previously non-pathogenic *E. coli* bacteria [32,33], which naturally occur in the human gut, the detection of these genes might be difficult and give false positive results, as nonspecifically-primed reactions may occur [34,35]. Keeping this in mind, we decided to check how the proposed method works in a situation with increased risk of nonspecific primer binding. For that, we used DNA isolated from an EDL933W strain that is *stx*-positive (+) and DNA from an *stx*-negative (−) *E. coli* K12 MG1655 strain as the representative of non-pathogenic bacteria. DNA was used in different amounts: 1, 10 and 100 pg, prepared as ten-fold dilutions of 1 ng of genomic DNA, which corresponds to 168, 1680 and 16,800 CFU, respectively. Additionally, we increased the number of PCR cycles to 40, as excessive cycling increases the opportunity for nonspecific amplification [36,37]. As expected, analysis of PCR products by agarose gel electrophoresis allowed us to identify smeared and nonspecific bands, especially occurring in the case of *stx2* detection (Figure 3). Note that the similar problem with nonspecific binding during PCR-based detection of the *stx2* gene was also described previously by Fagan and collaborators [34]. Importantly, even in the case of the appearance of nonspecific PCR products (which is likely to occur during testing of previously unknown, natural isolates of bacteria), the fluorescence is not detected over a UV transilluminator, contrary to the presence of DNA bands and smears on an electropherogram (Figure 3). Post-PCR visual observation of the fluorescence directly in the tube that we propose here allows avoidance of false positive results in dubious situations when a nonspecific PCR product is similar in size to that of a target amplicon (Figure 3, Lane 12). The specificity of the presented method is increased in comparison to conventional PCR because of the probe, which requires an additional complementary region within the template DNA. This may indicate that interpretation of the results of the test performed according to the proposed procedure is easier than that of the traditional PCR-based assay, and thus, our method is more specific, while, as indicated in Figure 3, its sensitivity is at a similar level, equal to 10 pg for both genes.

**Figure 4:**
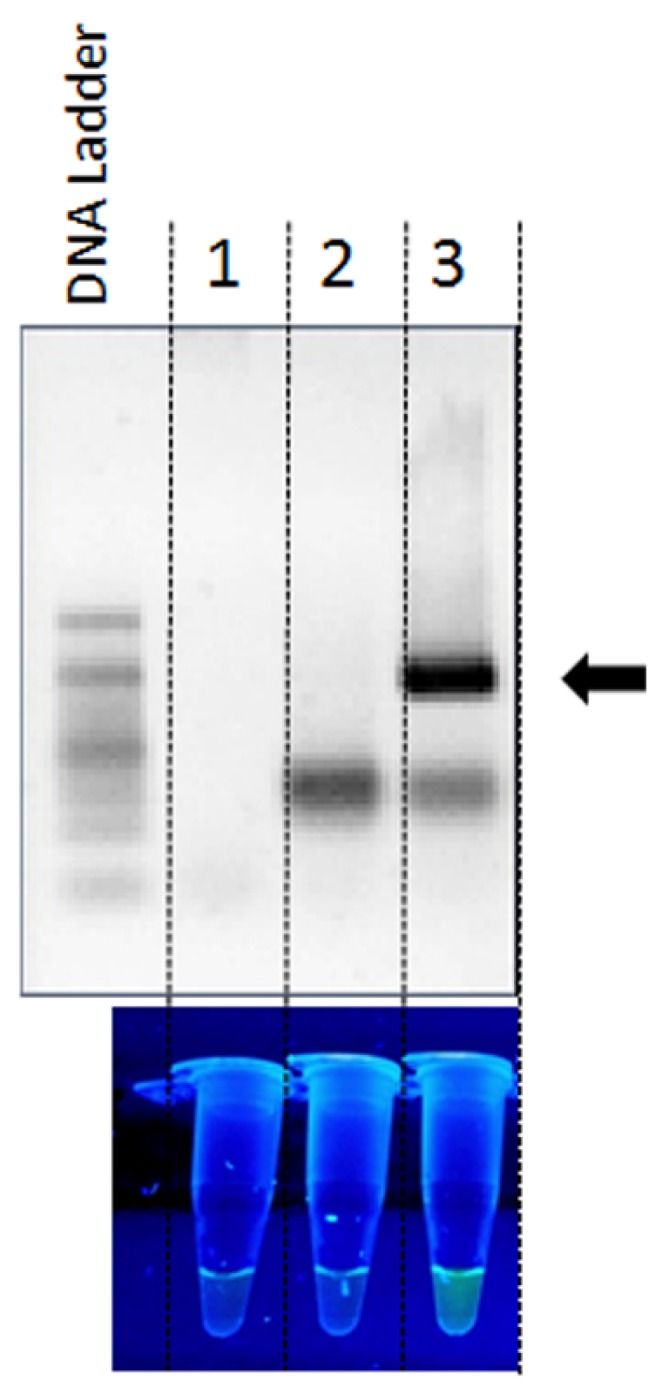
Analysis of the DNA from *Bartonella henselae* strain Houston-1 for the presence of the sequence encoding 16S rRNA, using PCR amplification with a FAM- and BHQ-1-labelled probe. Detection of the signal from the 16SrRNABhprobe complementary to the gene encoding 16S rRNA was performed by analysis of PCR products by agarose gel electrophoresis and using a UV transilluminator. The control reactions were performed without the primers (Lane 1) or DNA template (Lane 2). The target PCR product size of 180 bp is indicated by the black arrow (Lane 3).

The total time required for the procedure proposed in this report is as short as 1.5 h, including genomic DNA isolation and PCR amplification with simultaneous annealing and extension steps (see the Experimental Section for details). As the detection method is based on a simple PCR reaction, the only special equipment necessary to follow it consists of a thermocycler and a UV transilluminator, which are usually available in most laboratories (including those located in provincial hospitals), so it can be easily applied for a rapid, preliminary detection of not only STEC bacteria, but also other pathogens. Following this, we found that this procedure may be used in the detection of tick-transmitted bacteria. We performed a PCR amplification with primers and a probe that are complementary to the sequence encoding 16S rRNA of *Bartonella* *henselae* under the same conditions as those used for the detection of *stx* genes (Figure 4, Lane 3). Again, a signal was detected only in the presence of *B. henselae* DNA, indicating the specificity of the assay (Figure 4). 

As described previously [38,39], non-specificity is a serious problem of PCR-based detection of 16S rRNA sequences of bacteria that are transmitted by ticks. Massung and Slater [38] indicated that one of the PCR based-assays they evaluated, designed for the detection of the 16S rRNA gene of *Anaplasma phagocytophilum,* the human granulocytic ehrlichiosis (HGE) agent, may also amplify 16S rRNA of other tick bacteria: *Rickettsia rickettsii* and *Bartonella henselae*, the non-HGE agents. Similarly to the difficulties described above with false positive detection of STEC infections, such a scenario may result in a false diagnosis of HGE infection and an inappropriate medical treatment in both situations. Hence, there is a need to improve the specificity of PCR-based detection assays, and we believe that our method (as an alternative to standard PCR or its supplementation) could be a solution to this problem. 

In summary, we propose a simple, rapid and inexpensive procedure for the detection of a specific fluorescence signal after the PCR reaction with primers designed for amplification of a particular DNA fragment. As examples for its usefulness, we have demonstrated that this method is suitable for identification of the presence of STEC and *B. henselae*. 

## 3. Experimental Section 

### 3.1. Bacterial Strains

*Escherichia coli* strains were obtained from the collections of the National Institute of Public Health-National Institute of Hygiene (Warsaw, Poland) and the Department of Molecular Biology of the University of Gdańsk (Poland). The characteristics of all strains are provided in Table 1. STEC strains were identified by using the commonly-accepted methods (“gold standards”), *i.e.*, by employing a commercially-available cytotoxicity test on the Vero cell line [40] or the commercially-available test for the detection of verotoxigenic *E. coli* based on the reversed passive latex agglutination technique VTEC-RPLA assay (Oxoid Ltd., Basingstoke, UK), as described previously [41]. Additionally, the results were confirmed by a PCR-based method [42,43].

### 3.2. DNA Amplification and Detection of Signals Specific for Particular DNA Sequences

Genomic DNA from bacterial strains was isolated either by a boiling method [44] or in accordance to a method described previously [45]. Genomic DNA from *Bartonella henselae* strain Houston-1 (No 49882D-5) was provided by American Type Culture Collection ATCC (Manassas, VA, USA. DNA concentration was determined using a NanoDrop 1000 purchased from Thermo Fisher Scientific (Waltham, MA, USA). CFU values were established on the basis of genome copy numbers, which were calculated using a program available at the website: http://cels.uri.edu/gsc/cndna.html.

Primers used in PCR reactions were as follows: tox1F (5′-GAC GAT ACC TTT ACA GTT AAA GTG GGT-3′) and tox1R (5′-TCT CCG CCT GCT ATT TTC ACT-3′) for *stx1* (coding for Shiga toxin 1), tox2F (5′-TTC CAA GTA TAA TGA GGA TGA CAC A-3′) and tox2R (5′-CCC ACA TAC CAC GAA TCA GGT-3’) for *stx2* (coding for Shiga toxin 2), 16SrRNABhF (5′-CGT CAG TAA TGG ACC AGT GAG CC-3′) and 16SrRNABhR (5′-GCA TGT AGG ATA TTT AAG TCA GAG-3′). The length of reaction products was 196 and 211 bp, for *stx1* and *stx2*, respectively, and 180 bp for 16S rRNA. The probes, added to PCR reactions at a concentration of 150 nM, were labeled at the 5′ end with the fluorescent agent FAM (6-carboxylfluorescein) and at the 3′ end with the fluorescence quencher BHQ-1. The nucleotide sequences of the probes were as follows: tox1probe (for *stx1*) 5′-(FAM)-AAT CTT CAG TCT CTT CTT CTC AGT GCG CAA AT-(BHQ-1)-3′; tox2probe (for *stx2*) 5′-(FAM)-AAT CTG CAA CCG TTA CTG CAA AGT GCT CA-(BHQ-1)-3′; and 16SrRNABhprobe (for 16S rRNA of *Bartonella henselae*) 5′-(FAM)-TAC CTC TAC ACT CAG AAT TCC ACT CAC CTC TTC CA-(BHQ-1)-3′. The sequences of these probes were designed in this study, and the modified probes were purchased from Oligo.pl (Warsaw, Poland) and Sigma Aldrich (St. Louis, MO, USA).

The PCR reactions were performed with the use of Taq polymerase, employing the DFS-Taq DNA Polymerase kit (BIORON GmbH, Ludwigshafen, Germany) and dNTPs (Thermo Fisher Scientific, Waltham, MA, USA), under conditions recommended by the manufacturers and using the following program: 2 min initial denaturation at 95 °C, followed by 28 (or 40 in the experiment from Figure 3) cycles of denaturation for 15 s and simultaneous annealing and extension for 1 min at 60 °C. In the presence of *stx1* or *stx2* in the genome of *E. coli* strain being tested or the 16S rRNA sequence in the genomic DNA of *Bartonella henselae*, fluorescence appeared upon completion of the PCR reaction. This fluorescence was observed in a dark room over a UV transilluminator (Vilber Lourmat, Marne-la-Vallѐe, France), and photos were taken using a Panasonic Lumix DMC-FX60 digital camera (Panasonic, Osaka, Japan). Measurements of the fluorescence signal at 485/535 nm were performed using a VIKTOR 1420 plate reader (Perkin Elmer, Waltham, MA, USA). PCR amplification products were separated via 2% agarose (Prona, Spain) gel electrophoresis, performed at 100 V and visualized after staining with ethidium bromide (Sigma Aldrich, St. Louis, MO, USA), using the Gel Doc XR gel documentation system (Bio-Rad Laboratories, Hercules, CA, USA).

## 4. Conclusions

A new, specific, rapid and inexpensive procedure for the identification of bacteria carrying pathogenic genes has been developed. This method is based on the detection of the fluorescent signal under UV light, directly in the reaction tube.
